# Childhood glaucoma profile in a tertiary centre in Egypt according to the childhood glaucoma research network classification

**DOI:** 10.1371/journal.pone.0279874

**Published:** 2023-01-13

**Authors:** Yasmine M. El Sayed, Abdelrahman M. Elhusseiny, Ghada I. Gawdat, Amanne F. Esmael, Hala M. Elhilali

**Affiliations:** Department of Ophthalmology, Kasr Al-Ainy Hospitals, Cairo University, Giza, Egypt; The University of Iowa, UNITED STATES

## Abstract

**Purpose:**

To describe the prevalence and clinical characteristics of a large cohort of childhood glaucoma patients that presented to a tertiary Egyptian children’s hospital using the childhood glaucoma research network (CGRN) classification.

**Methods:**

A retrospective review of the medical records of all patients ≤ 14 years with a diagnosis of childhood glaucoma or glaucoma suspects who presented to Children’s Hospital between January 2014 to December 2019 was conducted. Data collected included age at the time of diagnosis, gender, laterality, prenatal history, parental history, including consanguinity, intraocular pressure, horizontal corneal diameter, and cup-to-disc ratio.

**Results:**

A total of 1113 eyes of 652 patients with diagnoses of either childhood glaucoma or glaucoma suspects were included in the study. Six hundred and sixteen patients (94%) were born full-term. A history of positive parental consanguinity was identified in 334 patients (51.2%). Almost 60% of patients were males. Primary congenital glaucoma (PCG) was the most prevalent diagnosis (68.2%), followed by glaucoma suspects (10.4%) and glaucoma following cataract surgery (GFCS) (8.4%). Juvenile open-angle glaucoma was the least prevalent category (0.3%). Other categories including glaucoma associated with non-acquired systemic disease, glaucoma associated with non-acquired ocular disease, and glaucoma associated with acquired conditions represented 5.8%, 4.7%, and 1.9%, respectively.

**Conclusions:**

PCG is the most common form of glaucoma in Egypt. More than half of the pediatric glaucoma patients had a positive history of parents’ consanguinity.

## Introduction

Childhood glaucoma is a heterogeneous group of disorders characterized by ocular hypertension which, if left untreated, results in optic nerve damage and visual loss. Glaucoma, which affects more than 300,000 children worldwide, is responsible for 5% of blindness in the pediatric age group [1]. Multicentre studies looking at the different causes and types of glaucoma, can help better the understanding of disease subtypes, as well as enable the development of preferred practice patterns for the management of each. This requires a unified classification for childhood glaucoma worldwide, and although several classification schemes have been developed [2–4], none of them were reproducible enough to become widely adopted. More recently, the Childhood Glaucoma Research Network (CGRN) proposed a new classification system, which categorizes childhood glaucoma into seven subtypes, allowing for a more straightforward categorization of pediatric glaucoma cases. The classification, proposed by an international consortium of glaucoma specialists, was validated at the World Glaucoma Association (WGA) in 2013, making it the first International Consensus Classification of childhood glaucoma [5].

In this study, we describe the clinical characteristics of pediatric glaucoma in patients presenting to the pediatric ophthalmology department, the main tertiary referral center in Egypt. The aim of the study was to analyze the demographics, patterns, and presentation of childhood glaucoma in our population as well as to evaluate the CGRN classification as a tool that can assist in planning collaborative research and allow us to gain a better understanding of the various subtypes of this disorder.

## Materials and methods

This was a retrospective study that included all patients ≤ 14 years (age limit at our hospital) with a diagnosis of pediatric glaucoma or glaucoma suspects who presented to the Pediatric ophthalmology unit at Children’s Hospital during the period between January 2014 to December 2019. Patients with incomplete data or those with only one clinic visit data were excluded from the study. Data collected included age at the time of diagnosis, gender, laterality, ethnicity, type of glaucoma, birth history, family history including parental consanguinity, lens status, intraocular pressure (IOP), horizontal corneal diameter (HCD), cup-to-disc ratio (C/D), central corneal thickness (CCT) and slit-lamp findings such as Haab striae. IOP was measured using Perkins® applanation tonometry. Chloral hydrate sedation was used in younger patients when the ocular examination was difficult to perform. The CCT measurements were taken using the ultrasound-based DGH 55 handheld Pachmate (DGH Technology Inc, Exton, PA) either during an office visit, with the patient awake or sedated by chloral hydrate, or with the patient under general anesthesia. Patients were classified into one of seven categories according to CGRN classification [6]. Categories included 1) Primary congenital glaucoma (PCG), 2) Juvenile open-angle glaucoma (JOAG), 3) Glaucoma following cataract surgery (GFCS), 4) glaucoma associated with non-acquired systemic disease or syndrome (e.g., Sturge-Weber syndrome (SWS), Dandy-Walker syndrome, and mucopolysaccharidosis), 5) glaucoma associated with non-acquired ocular disease (e.g., aniridia, sclerocornea, and Peters anomaly), 6) glaucoma associated with acquired conditions (e.g., trauma, uveitis, and steroid-induced glaucoma), and 7) glaucoma suspects. PCG was further classified according to the age of onset into either neonatal (0–1 months), infantile (>1–24 months) or late onset PCG (>24 months). Glaucoma was diagnosed by ≥ 2 of the following clinical findings: 1) IOP more than 21 mmHg, 2) optic nerve damage as evidenced by glaucomatous cupping or C/D asymmetry between both eyes of 0.2 or more or focal thinning, 3) anterior segment findings such as Haab striae or large corneal diameter, 4) glaucomatous visual field defect in older children, 5) increased axial length or progressive myopia. Glaucoma suspects were diagnosed by one or more of the following criteria: 1) optic nerve appearance suspicious of glaucoma, 2) visual field defects suspicious of glaucoma, 3) ocular hypertension (IOP > 21 mmHg on 2 separate occasions) with normal optic nerve and normal visual field, 4) increased axial length with normal IOP, and 5) increased corneal diameter with normal IOP [6].

The study was approved by the ethics committee and adhered to the tenets of the declaration of Helsinki. Surgical consent was obtained from all patients, but informed consent to publish the current study was waived by the ethics committee since the risk to the patient was minimal, and obtaining consent would be impractical.

### Statistical analysis

Data were entered using Microsoft Excel 2016 and analyzed using statistical package for social sciences (SPSS Inc., Chicago, IL, USA, version 24). Quantitative data were expressed as median, mean ± standard deviation (SD), and range. Bivariate relationships were displayed in cross-tabulations and a comparison of proportions was performed using the chi-square or Fisher’s exact tests where appropriate. A p-value less than 0.05 was considered statistically significant. Kolmogorov-Smirnov and Shapiro-Wilk tests measured the normality of the data.

Multivariate analysis was performed with the type of glaucoma as a dependent variable and baseline clinical characteristics as independent variables. A backward stepwise logistic regression was used to identify possible predictors of glaucoma type out of the following candidate variables: age, consanguinity, bilaterality, corneal clarity, family history, and the final outcome. At each step, variables were chosen based on p-values, with a p-value threshold of 0.05 used as the elimination criterion in the model. Starting with 6 candidate variables, a backward stepwise logistic regression model was used to reduce them to 5 which were age, consanguinity, bilaterality, corneal clarity, and family history.

## Results

**Fig 1:**
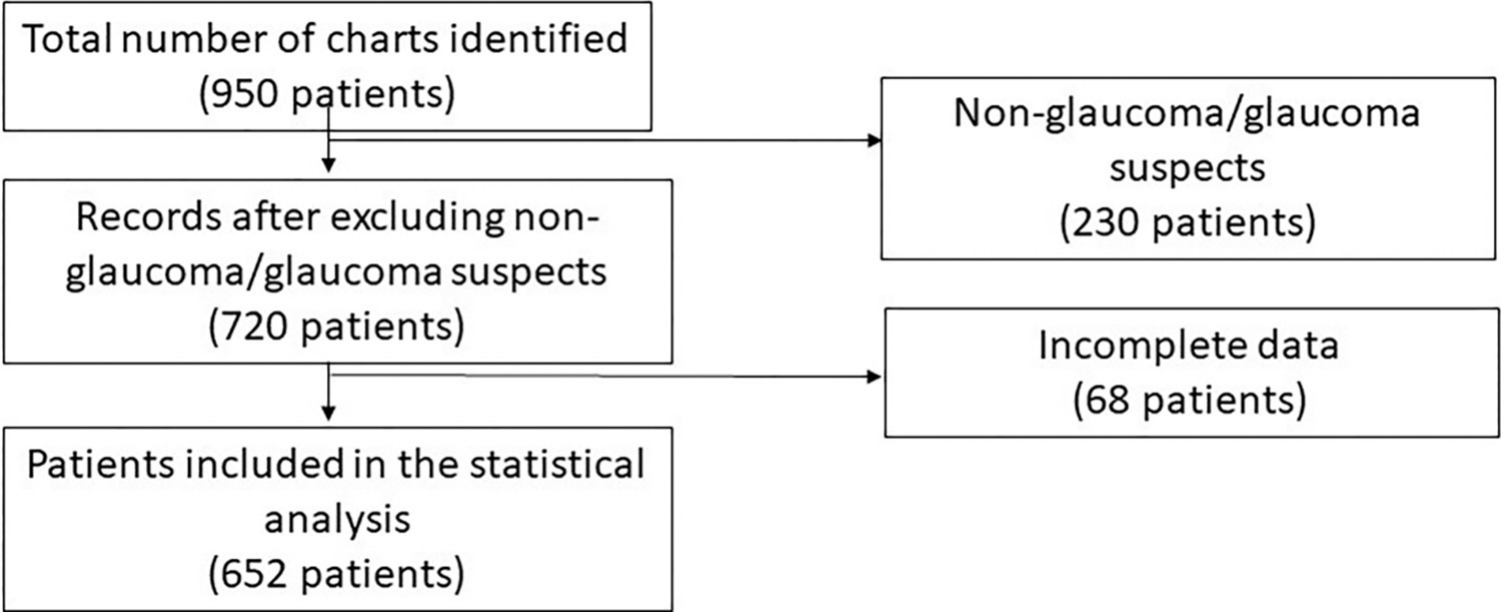
Flow-chart of the included patients.

A total of 720 patients were identified during the study period. Sixty-eight patients were excluded due to incomplete data (Fig 1). The current study included 1113 eyes of 652 patients with a diagnosis of pediatric glaucoma or glaucoma suspects. Table 1 shows the initial presenting symptoms of the glaucoma cohort. Corneal clouding, alone or with other symptoms, was the most common reason to seek medical care (40.8%), followed by a large or enlarging cornea (27.6%).

**Table 1:** Initial presenting symptom/sign(s) of the glaucoma cohort.

Presenting manifestation	Number of eyes (%)
Cloudy cornea only	314 (28.2%)
Referral	193 (17.3%)
Large cornea	180 (16.1%)
Lacrimation	84 (7.5%)
Cloudy cornea + Lacrimation	81 (7.2%)
Accidental diagnosis	77 (6.9%)
Large cornea + photophobia	68 (6.1%)
Cloudy cornea + Large cornea	60 (5.3%)
Photophobia	35 (3.1%)
Nystagmus	21 (0.18%)
Total cohort	1113 (100%)

The most prevalent type of glaucoma in our cohort was PCG (445 patients, 68.2%), followed by glaucoma suspects (68 patients, 10.4%) and GFCS (55 patients, 8.4%). Juvenile open-angle glaucoma was the least prevalent category (2 patients, 0.3%). Other categories including glaucoma associated with non-acquired systemic disease, glaucoma associated with non-acquired ocular disease, and glaucoma associated with acquired conditions represented 5.8% (38 patients), 4.7% (31 patients), and 1.9% (13 patients), respectively.

According to the age of onset, PCG was further classified into either neonatal onset PCG (414 eyes, 52.1%), infantile-onset PCG (347 eyes, 43.7%), or late-onset PCG (33 eyes, 4.1%). The mean age of onset of PCG was 0.19±0.3 months (median: 0.1 months, range: birth-1 month) in the neonatal PCG compared to 6.4±8.7 months (median: 9 months, range: 45 days- 24 months) in the infantile group and 48.3 ±13.7 months (median: 53 months, range: 26 months-120 months) in the late-onset PCG. The mean time between the onset of symptoms and presentation to our institute was 3.2±2.5 months (median: 5 months, range; 3 days- 27 months). After excluding patients that had glaucoma surgeries in other hospitals before presenting to us, the mean time for seeking medical care was 2.3±0.9 months (median: 4.2 months).

Table 2 summarizes the causes of secondary glaucoma in our cohort. In GFCS, 46 eyes were aphakic and 39 eyes were pseudophakic. The most common systemic syndrome associated with glaucoma was SWS (15 eyes) while the most common ocular condition associated with glaucoma was aniridia (10 eyes).

**Table 2:** Causes of secondary glaucoma.

Type of glaucoma	Cause (number of eyes)
Glaucoma following cataract surgery	• Pseudophakic (39) • Aphakic (46)
Glaucoma associated with non-acquired systemic disease	• Sturge-Weber syndrome (15) • Neurofibromatosis-1 (5) • Down syndrome (8) • Mucopolysaccharidosis (4) • Dandy-Walker syndrome (2) • Stickler syndrome (2) • Ichthyosis (4) • Chromosomal disorder (9) • Congenital rubella (4) • Noonan syndrome (5)
Glaucoma associated with non-acquired ocular disease	• Aniridia (10) • Peter’s anomaly (8) • Sclerocornea (6) • Persistent fetal vasculature (3) • Axenfeld- Rieger syndrome (5) • Iris dysgenesis (3) • Microcornea (2) • Ocular melanosis (1) • Microspherophakia (6) • Ectopia Lentis (1)
Glaucoma associated with acquired conditions	• Traumatic (8) • Steroid-induced (3) • Uveitic (4)

Table 3 summarizes the baseline and clinical characteristics of each of the 7 groups. Most patients (94.4%) were born full-term. A stepwise backward logistic regression found that younger age was significantly associated with PCG (p<0.0001). Additionally, positive parental consanguinity (p = 0.02), bilateral disease (p = 0.006), positive family history (p = 0.019), and corneal haziness (p = 0.026) were associated with PCG diagnosis.

**Table 3:** Baseline characteristics of the whole cohort.

Type of glaucoma	Number of patients (eyes)	Full term pregnancy	Parental consanguinity	Family history of childhood glaucoma	Mean age at time of diagnosis (months)	Bilateral	Right eye involvement	Male patients	Mean IOP at initial presentation (mmHg)	Mean HCD at presentation (mm)	Mean C/D ratio at initial presentation
**PCG**	445 (794)	430/445 (96.6%)	256/445 (57.5%)	35/445 (7.8%)	5.8±15.7 (median: 1 month)	349/445 (78.4%)	396 (49.8%)	272/445 (61.1%)	23.2±7.5 (median: 22 mmHg)	13.3±1.2 (median: 13 mm)	0.6±0.2 (median: 0.6)
**JOAG**	2 (4)	2/2 (100%)	1/2 (50%)	1/2 (50%)	120±12.1 (median: 84 months)	2/2 (100%)	2 eyes (50%)	2/2 (100%)	23±2.8 (median: 25 mmHg)	12±0 (median: 12 mm)	0.56±0.09 (median: 0.5)
**GFCS**	55 (85)	46/55 (83.6%)	22/55 (40%)	4/55 (7.2%)	53.6±42.1 (median: 48 months)	30/55 (54.5%)	42 (49.4%)	28/55 (50.9%)	24.7±8.4 (median: 26 mmHg)	12.6±3.7 (median: 10 mm)	0.59±0.2(median: 0.6)
**Glaucoma associated with non-acquired systemic disease or disorder**	38 (58)	36/38 (94.7%)	14/38 (36.8%)	3/38 (7.8%)	26.9±41.1 (median: 1 month)	22/38 (57.8)	31 (53.4%)	23/38 (60.5%)	24.1±8.6 (median: 22 mmHg)	12.1±1.6 (median: 13 mm)	0.57±0.2 (median: 0.6)
**Glaucoma associated with non-acquired ocular anomalies**	31 (45)	30/31 (96.7%)	15/31 (48.3%)	4/31 (12.9%)	17.5±29.6 (median: 1 month)	15/31 (48.3%)	25 (55.5%)	16/31 (51.6%)	21.7±8.6 (median:19.5)	12.7±0.5 (median: 12.7)	0.65±0.2 (median: 0.7)
**Glaucoma associated with acquired conditions**	13 (15)	11/13 (84.6%)	1/15 (6.6%)	0/13 (0%)	60±42.2 (median: 48 months)	2/13 (15.3%)	8 (53.3%)	7/13 (53.8%)	24.9±8 (median: 24 mmHg)	12±0.5 (median:12 mm)	0.7±0.1 (median: 0.7)
**Glaucoma suspects**	68 (112)	61/68 (89.7%)	25/68 (36.7%)	6/68 (8.8%)	38±50.7 (median: 30 months)	44/68 (64.7%)	54 (48.2%)	42/68 (61.7%)	15.3±5.3 (without glaucoma medications) (median: 14.5)	11.5±1.5 (median: 11)	0.45±0.2 (median:0.4)
**Whole cohort**	652 (1113)	616/652 (94.4%)	334/652 (51.2%)	53/652 (8.1%)	14.1±30.3 (median: 2 months)	464/652 (71.1%)	558/1113 (50.1%)	390/652 (59.8%)	21.8±7.8 (median: 22)	12.9±1.5 (median: 12)	0.56±0.2 (median: 0.6)

### Laterality

Among the 652 patients, 464 (71.1%) had bilateral disease (p = 0.02). In the PCG group, 78.4% of patients had bilateral disease while in glaucoma associated with acquired disease only 2 patients had bilateral disease. The right eye was affected in 50.1% (558 eyes) (p = 0.1).

### Gender

Males were more affected in all of the 7 glaucoma groups, representing 59.8% of patients in the whole cohort, however, the difference did not reach statistical significance (p = 0.07).

### Parental consanguinity

Positive parental consanguinity was found in 57.7% of the PCG group and 40% of the GFCS group (p<0.001). Only one patient in the “glaucoma associated with acquired disease” group had a history of positive consanguinity. In addition, 26 of the 256 patients with a history of positive parental consanguinity in the PCG group also had a family history of childhood glaucoma.

### Age at the time of presentation

The mean age at the time of diagnosis was 14.1±30.3 months (median: 2 months, range; 6 days– 168 months) in the whole cohort. In the PCG group, the mean age at the time of presentation was 5.8±15.7 months (median: 1 month, range: birth-120 months) while in the JOAG group the mean age was 120±12.1 months (median: 84 months) (p<0.001).

### Ocular evaluation and management

In the glaucoma cohort, the mean IOP at initial presentation was 24.9±8.0 mmHg (median: 24 mmHg, range: 22–60 mmHg) on a mean of 1.3±0.5 glaucoma medications (median: 1 medication, range: 0–5 medications). In the whole cohort, the mean HCD was 12.9±1.5 mm (median: 13 mm, range: 10–18 mm) and the mean C/D ratio was 0.56±0.2 (median: 0.6, range: 0.1–1). The mean CCT was 655±153 μ (median:630 μ, range: 450–800 μ). Of the 584 glaucoma patients, 182 (31.1%) had a history of one or more glaucoma surgery before presenting to our hospital (Table 4). Of the 107 cases that had trabeculectomy before presenting to our hospital, 61 had it as an initial glaucoma procedure.

**Table 4:** Summary of glaucoma procedures performed in 182 glaucoma patients before presenting to our hospital.

Surgery	Number of surgeries performed
Goniotomy	30
Trabeculotomy	55
Trabeculectomy	107
Combined trabeculotomy-trabeculectomy	6
Glaucoma drainage device	12
Cyclophotocoagulation	23
Unknown surgery	39
Total number of surgeries	272

In the glaucoma suspects group, the most common presentation was optic nerve cupping in the setting of normal IOP (Table 5). The mean IOP at the initial presentation of the suspects group was 15.3±5.3 mmHg (median: 14.5 mmHg, range:10–30 mmHg). We excluded 48 eyes previously documented as glaucoma suspects because they did not conform with the CGRN classification. Examples include IOP>21 mmHg documented only once, aniridia, Peters anomaly, the fellow eye of PCG, and sibling of PCG patient.

**Table 5:** Initial presentations of glaucoma suspects in our cohort.

Presentation	Number of eyes (%)
Optic nerve cupping	54 (48%)
Large corneal diameter	28 (25%)
IOP > 21 mmHg on 2 separate occasions	16 (14%)
Large corneal diameter and cupping	10 (9%)
Large corneal diameter and cloudy cornea	4 (4%)
Visual field defects suspicious of glaucoma	0 (0%)
Increased axial length with normal IOP	0 (0%)
Total	112 (100%)

IOP: Intraocular pressure.

## Discussion

Standardizing the classification of childhood glaucoma is the first step toward planning longitudinal surveys on its outcomes, recommending management guidelines for subtypes of glaucoma as well as facilitating collaborative, multicentre research in this field. We found the CGRN classification to be a specific and reproducible way to systematically classify childhood glaucoma patients. Reclassifying patients according to the 7 categories was simple and logical: no patients had overlapping diagnoses.

Data from the 652 patients included in our study were consistent with the literature, concerning male predominance [7–9], bilaterality [10,11] and the high incidence of parental consanguinity [12,13]. Although statistically insignificant, male predominance was noted in all subtypes of glaucoma in our cohort. The exact reason for this observation is unknown, however, it may be related to societal or cultural norms where male children are getting more attention and families bring them for getting the treatment. Our results are also in agreement with other studies that reported PCG as the most common childhood glaucoma [8–10]. The high prevalence of PCG in our cohort (68%) is most likely caused by the genetically inbred populations in rural areas of Egypt in which parental consanguinity is relatively common. Most of our PCG patients had consanguineous parents (57%), and 26 out of the 256 patients with consanguineous parents had a family history of PCG, mostly among their siblings. This suggests around a 10% chance of siblings of PCG patients developing glaucoma if their parents are relatives. On the other hand, out of 189 PCG patients with non-consanguineous parents, 9 (5%) had a positive family history of childhood glaucoma, with none being in their siblings. This suggests that non-consanguineous parents have a negligible chance of having a second child with PCG. Ethnicity could be another contributing factor to the high incidence and prevalence of PCG in Egypt. The British Infantile and Childhood Glaucoma (BIG) eye study reported the incidence of PCG among children of Pakistani origin to be 9 times that in Caucasians, while the reported incidence in the Middle East is 1:2500 live births compared to 1:18500 in Great Britain [14]. The male predominance observed in our PCG patients is also in agreement with most reports in the literature. The genetic basis for this sex predilection is still unknown and despite the recent major advances in the genetics of PCG, we do not yet have an explanation for why certain populations, like South India [10], do not exhibit the same male predilection among their PCG patients.

The age of onset of glaucoma in our cohort was 14.1±30.3 months, which is lower than previously reported in the literature [7,9]. Likewise, this can be explained by the high prevalence of PCG among our cases, with more than half of our PCG patients presenting in the first month of life (52%). This is followed by infantile-onset cases (aged >1–24 months) which represented 44% of PCG patients. This is in contrast to the findings of other studies that report a higher incidence of infantile compared to neonatal subtypes of PCG [15]. The relatively earlier onset of PCG in our cohort could be related to the high rate of consanguinity, which can result in a more aggressive disease that often presents as early as the first month of life [16,17]. Further molecular studies may help reveal additional factors that influence the expression of the PCG phenotype.

**Fig 2:**
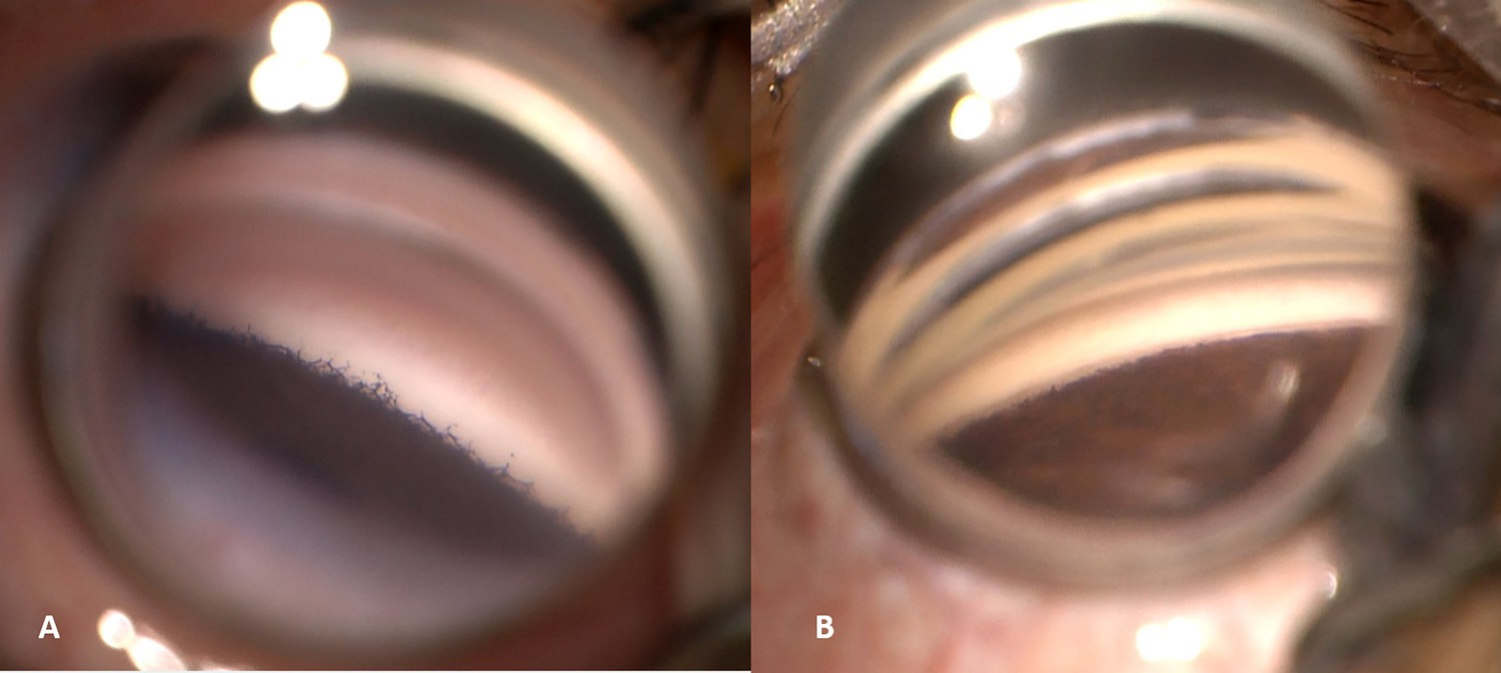
Angle appearance in two PCG patients **A.** The angle showing high iris insertion is characteristic of a “premature” angle. **B.** Apparently normal angle appearance is indistinguishable from JOAG and primary open angle glaucoma cases.

JOAG was the least prevalent subtype in our cohort with only 2 patients reported (0.3%). This is different from other studies on childhood glaucoma which reported an incidence of 7–16% [7,9,10]. This can be partially explained by the fact that our pediatric hospital sees children aged 14 years or less, with older children being seen in the adult clinics of the hospital. Another reason could be the overlap between the diagnosis of JOAG and late-onset PCG, where JOAG has a normal-looking angle while PCG usually has distinctive angle features sometimes exhibiting high iris insertion. Categorizing primary childhood glaucoma after the age of 2 years into late-onset PCG and JOAG according to the CGRN classification may help in studying each of these categories separately, enabling us to reach specific treatment guidelines for each. In our cohort, late-onset PCG represented 4.1% of PCG cases and although gonioscopy was performed intraoperatively for most of these cases, a description of the angle was rarely documented in the patient’s notes. Nevertheless, some patients with PCG, whether late- or early-onset, still exhibit apparently normal-looking angles that can be difficult to differentiate from those of JOAG cases (Fig 2). The only way to differentiate between such cases would probably be through genetic studies.

GFCS represented 8.4% of glaucomatous eyes. The incidence of glaucoma following infantile cataract removal varies according to the definition of glaucoma and the duration of follow-up in the different studies, with a reported annual incidence of 5.25% in the first 7 years after lens extraction [18]. The 10-year incidence was reported by the Infant Aphakia Treatment (IATS) group to be 22% and although it was previously believed that aphakic eyes were more likely to develop secondary glaucoma than pseudophakic eyes, more recent evidence from IATS and other studies [19,20] showed that having an intraocular lens was not protective against glaucoma. In our study, there was no significant difference in the number of aphakic (46 eyes) and pseudophakic eyes (39 eyes) that developed glaucoma. The CGRN network classification subcategorized GFCS into open angle (≥ 50% open) and angle closure glaucoma (<50% open). One drawback we found in our documentation of childhood glaucoma cases was that gonioscopy was not systematically performed for eyes with GFCS. Unless the case had obvious secondary angle-closure glaucoma, such as in the presence of iris bombe or iridocorneal adhesions, it was not documented in clinic notes whether this was an angle closure or open-angle glaucoma. In consideration of this, we intend to add gonioscopy and/or anterior segment OCT in the assessment of our cases in the future, as this can help better address the underlying cause of IOP elevation, in addition to evaluating the outcomes of treatment options like angle surgery in aphakic and pseudophakic glaucoma patients [21].

The prevalence of glaucoma suspects in our study was only 10% which is less than previously reported in other studies that followed the CGRN classification for childhood glaucoma [9,11]. This is most likely caused by referral bias, with our hospital being a tertiary referral center for surgical management of childhood glaucoma. The most common cause was the presence of a suspicious optic disc appearance (48%). This is consistent with findings by Hoguet et al and Bouhenni et al, although in both studies the second cause for glaucoma suspicion was a high IOP whereas in our study it was the presence of a large corneal diameter.

We did not find a preponderance of right eye involvement in patients with glaucoma associated with nonacquired systemic disease, unlike what was previously reported by Hoguet et al [9]. Although in both our study and Hoguet’s, phacomatoses particularly SWS were the most common causes of glaucoma associated with nonacquired systemic disease, yet in their study, all unilateral cases (9 patients) had glaucoma in their right eye, compared to 9/16 unilateral cases in our study. This can be caused by racial or genetic factors which need to be determined in a larger number of patients.

The main strength of our study lies in the large number of patients referred from different regions of the country to our tertiary referral center, thus representing diverse sectors of the Egyptian population. Yet the study has several limitations inherent to retrospective studies and including patients only below the age of 15 because of our hospital policy. Gonioscopy was missing from most patients’ notes restricting us from drawing conclusions on the frequency of closed-angle compared to open-angle glaucoma, especially in GFCS. Data on visual acuity were missing owing to the young age of most patients at the presentation. Axial length was not routinely documented. Many cases with ocular hypertension secondary to uveitis or following cataract surgery may have been missed, being mostly managed in the pediatric uveitis and cataract clinics of our hospital. Finally, the number of glaucoma suspects does not project the real prevalence of these cases among our childhood glaucoma population, as a large percentage is managed either by primary care physicians outside our hospital or non-glaucoma specialists in our pediatric ophthalmology department and only referred unless there is a high index of suspicion for glaucoma. Due to the relative rarity of childhood glaucoma, the development of international registries is essential to conducting larger studies evaluating the natural history of the disease and the outcomes of different therapeutic interventions. Investigating the socioeconomic factors related to the care of childhood glaucoma is needed.

In conclusion, the CGRN classification provides a simple and logical way to categorize pediatric glaucoma cases, which can facilitate the development of collaborative research into these rare groups of disorders.
